# REF1 peptides significantly enhance transformation efficiency but inhibit shoot regeneration in citrus *agrobacterium*-mediated transformation

**DOI:** 10.1093/hr/uhag134

**Published:** 2026-04-07

**Authors:** Yu Feng, Wenting Wang, Yuanchun Wang, Xiaoen Huang, Jin Xu, Nian Wang

**Affiliations:** Citrus Research and Education Center, Department of Microbiology and Cell Science, Institute of Food and Agricultural Sciences (IFAS), University of Florida, Lake Alfred, FL 33850, U.S.A; Citrus Research and Education Center, Department of Microbiology and Cell Science, Institute of Food and Agricultural Sciences (IFAS), University of Florida, Lake Alfred, FL 33850, U.S.A; Citrus Research and Education Center, Department of Microbiology and Cell Science, Institute of Food and Agricultural Sciences (IFAS), University of Florida, Lake Alfred, FL 33850, U.S.A; Citrus Research and Education Center, Department of Microbiology and Cell Science, Institute of Food and Agricultural Sciences (IFAS), University of Florida, Lake Alfred, FL 33850, U.S.A; Citrus Research and Education Center, Department of Microbiology and Cell Science, Institute of Food and Agricultural Sciences (IFAS), University of Florida, Lake Alfred, FL 33850, U.S.A; Citrus Research and Education Center, Department of Microbiology and Cell Science, Institute of Food and Agricultural Sciences (IFAS), University of Florida, Lake Alfred, FL 33850, U.S.A

## Abstract

*Agrobacterium*-mediated transformation remains challenging for economically important citrus crops because of low transformation and regeneration efficiencies. REGENERATION FACTOR1 (REF1), a plant elicitor peptide, is a local wound signal perceived by PROPEP RECEPTOR-LIKE KINASE 1 (PORK1) and transduced via WOUND-INDUCED DEDIFFERENTIATION 1 (WIND1). Exogenous REF1 peptide application has recently been reported to improve transformation and regeneration of herbaceous plants including tomato, which was explored on *Agrobacterium*-mediated citrus transformation in this study. We identified REF1, PORK1, and WIND1 orthologs in sweet orange (*Citrus sinensis*), which encodes two REF1 isoforms, CsREF1–1 and CsREF1–2. REF1 of tomato (SlREF1) and the two CsREF1 isoforms of different concentrations were tested for their effect on *Agrobacterium*-mediated transformation of citrus via exogenous application. CsREF1–1 at 10 or 100 nM and CsREF1–2 at 100 nM significantly increased GFP-positive transgenic callus formation, whereas SlREF1 had no significant effect. In addition, CsREF1–1, CsREF1–2, and SlREF1 reduced shoot regeneration. To overcome this trade-off, we exposed epicotyl explants to 100 nM CsREF1–2 only during the first month and then transferred them to REF1-free regeneration medium, yielding 4.1-fold more GFP-positive shoots than the untreated control. Such a staged application of CsREF1–2 also improved GFP-positive shoot recovery in Carrizo citrange by 3.3-fold. Reverse transcription-quantitative PCR (RT-qPCR) showed that CsREF1–2 induced *CsWIND1* and *CsESR1,* which are critical for callus formation and shoot regeneration, but continuous CsREF1–2 exposure also suppressed shoot/meristem regulators, which was mitigated by removal of CsREF1–2 in the later stage. AlphaFold3 and PRODIGY predictions indicated the highest binding affinity for CsREF1–2 with CsPORK1, consistent with its superior activity. Overall, both CsREF1 peptides enhance citrus transformation but suppress regeneration, and staged exogenous application of CsREF1 peptides improves genetic improvement of recalcitrant perennial crops such as citrus, which decouples early dedifferentiation from later organogenesis, substantially expanding the toolbox for high-throughput functional genomics in citrus.

## Introduction

Citrus is among the most widely cultivated fruit crops worldwide, encompassing several economically important species, including sweet orange (*Citrus sinensis*), grapefruit (*C. paradisi*), and mandarin (*C. reticulata*), with sweet orange being the most widely planted [1]. Sweet orange is a natural hybrid between mandarin and pummelo (*C. maxima*) [2]. Most commercial citrus crops are **maintained as monocultures**, which lack genetic diversity and are therefore more vulnerable to both biotic and abiotic stresses [3, 4]. Citrus trees are susceptible to numerous diseases, including Huanglongbing (HLB), citrus canker, and other infectious diseases [5]. Additionally, they are exposed to major abiotic stresses, such as acidic, alkaline, and saline soils, drought, and heat stress, which are increasingly exacerbated by climate change [6]. These biological and environmental pressures underscore the urgent need for genetic improvement to enhance citrus resilience and productivity.

**Traditional breeding** in citrus trees **struggles** to meet these challenges due to their perennial nature, and distinctive reproductive biology [7]. Apomixis in citrus enables the asexual formation of seeds from maternal ovule tissues, bypassing meiosis and fertilization [8]. Consequently, offsprings inherit the **maternal genome**, making hybridization challenging. Most sweet orange cultivars are derived from somatic mutants and thus their background is monogenetic [2]. Additionally, citrus trees have a prolonged juvenile phase of 5–10 years, delaying fruit production, while their high heterozygosity complicates trait inheritance. Variability among nucellar seedlings results in unpredictable breeding outcomes, and male or female sterility further constrains traditional breeding efforts [9, 10].

*Agrobacterium*-mediated transformation is a potent tool in genetic improvement of citrus and other tree crops [11–13]. **However,** most citrus species are recalcitrant to *Agrobacterium*-mediated transformation. The first successful *Agrobacterium*-mediated transformation in *Citrus*  **spp.** was reported in 1990 using suspension cells, followed by transformations in epicotyl segments of **various** citrus species [14–16]. The transformation efficiency of a GUS expression vector **in sweet orange epicotyls ranged** from 1.54% to 6.08% [17]. Further advancements in gene silencing, transgenic overexpression and genome-editing tools have **contributed to** citrus **improvement**. In 2011, RNA silencing of the Citrus psorosis virus **(CPsV)** coat protein was employed to enhance resistance against CPsV while the overexpression of (E)-β-caryophyllene synthase reduced the attraction of psyllid (*Diaphorina citri*), the insect vector of HLB [18]. However, the positive plant regeneration efficiency was only approximately 5% [18]. In 2014, CRISPR-mediated genome editing was first used in citrus [19]. Multiple studies have further advanced citrus genome editing in grapefruit [20, 21], *Carrizo citrange* [*18**,*  *22**]*, sweet orang [12] and pummelo [20, 23], although the overall transformation efficiency remained low **about 5%** [**12****]**. More recently, a **transgene-free**  *Agrobacterium*-mediated epicotyl transformation approach was developed by **transient expression** of a **co-editing vector**, enabling the simultaneous editing of a herbicide-resistance gene and a targeted gene, with an efficiency of 1.9%. Furthermore, as **T-DNA** size increases, the efficiency of transformation significantly decreases [24, 25]. Thus, despite technological advances, the low efficiency of current transformation methods continues to constrain the application of CRISPR-based genome editing, RNA interference or overexpression strategies in citrus breeding.

**Recently, it was discovered that** wounding induces the expression of the Plant Elicitor Peptide **(PEP**), which **is** recognized by **PROPEP RECEPTOR-LIKE KINASE 1 (PORK1) in tomato. PEP–PORK1** signaling activates WIND1, promoting local defense responses and regeneration [26]. **Furthermore, a** 23-amino-acid segment of PEP, **termed** REGENERATION FACTOR1 (REF1), **is** recognized by PORK1 and **is sufficient to** accelerate transformation and regeneration not only in tomato but also in three important grain crops including soybean, maize, and wheat [26]. Despite these promising findings in herbaceous and annual crops, the effects of REF1 on tree crops including citrus remain unexplored.

In this study, we evaluated the effect of three different REF1 peptides on *Agrobacterium*-mediated epicotyl transformation of ‘Hamlin’ sweet orange. Three synthesized REF1 peptides, CsREF1–1 and CsREF1–2 from *C. sinensis* and SlREF1 from tomato (*Solanum lycopersicum*), were tested. Our results demonstrated that REF1 peptide(s) of citrus but not tomato significantly improved transformation efficiencies in citrus. In addition, CsREF1 peptides suppressed shoot regeneration. To address this, we conducted staged application of CsREF1 peptides to improve citrus transformation and regeneration by taking advantage of the transformation-promoting effects of REF1 while mitigating its inhibitory impact on shoot development.

## Results

### Identification of REF1, PORK1, and WIND1 homologs in citrus

**Figure 1:**
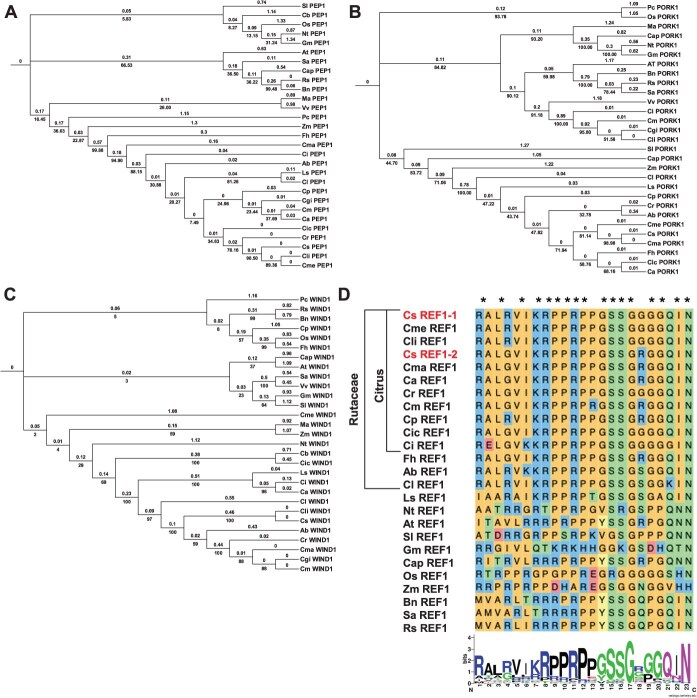
Phylogenetic analysis of PEP1, PORK1, and WIND1 proteins in Citrus and several other species. Phylogenetic tree of (A) PEP1, (B) PORK1, and (C) WIND1 protein sequences across citrus and relatives using the Neighbor-Joining method. (D) Multiple sequence alignment of the REF1 protein sequences using WebLogo. * indicates conserved amino acid in the Rutaceae family. Abbreviated species names are given before gene identifiers: *Citrus sinensis* (Cs), *Citrus medica* (Cme), *Citrus limon* (Cli), *Citrus maxima* (Cma), *Citrus aurantiifolia* (Ca), *Citrus reticulata* (Cr), *Citrus micrantha* (Cm), *Citrus paradisi* (Cp), *Citrus ichangensis* (Cic), *Citrus inodora* (Ci), *Fortunella hindsii* (Fh), *Atalantia buxifolia* (Ab), *Clausena lansium* (Cl), *Luffa scandens* (Ls), *Solanum lycopersicum* (Sl), *Glycine max* (Gm) *Mercurialis annua* (Ma), *Vitis vinifera* (Vv), *Capsicum baccatum* (Cb), *Nicotiana tabacum* (Nt), *Sinapis alba* (Sa), *Raphanus sativus* (Rs), *Brassica napus* (Bn), *Capsella rubella* (Cap), *Pyrus communis* (Pc) and *Arabidopsis thaliana* (At). The two different CsREF1s in *C. sinensis* were labeled in red.

Previous studies have shown that the REF1 peptide functions as a wound-induced signal that promotes plant transformation and regeneration through its receptor PORK1 and the downstream regulator WIND1 [26]. To identify whether a similar signaling module exists in citrus, BLASTp analyses using SlPEP (*Solyc04g072310*), SlPORK1(*Solyc03g123860*), and SlWIND1(*Solyc12g056980*) identified homologs in Citrus species (Fig. 1). The PEP (Fig. 1A), PORK1(Fig. 1B), and WIND1(Fig. 1C) proteins of Citrus species and relatives (*C. sinensis, C. medica, C. limon, C. maxima, C. aurantiifolia, C. reticulata, C. micrantha, C. paradisi, C. ichangensis, C. inodora, Fortunella hindsii, Atalantia buxifolia, Clausena lansium,* and *Luffa scandens*) shared 24%–44%, 36%–58%, 44%–47% identities and 51%–77%, 55%–71%, 53%–58% similarities, respectively, with their tomato counterparts. **Multiple sequence alignment of the 23 aa REF1 peptides** showed strong conservation among Rutaceae members and close relatives **(**Fig. 1D**)**. Several conserved residues, such as **R/P/G/N**, previously reported in tomato and *Arabidopsis* [*26**[,* were also detected in citrus REF1 peptides (Fig. 1D). Specifically, RPPRxGSSGxGGxIN were conserved in Rutaceae members (Fig. 1D). Across REF1 peptides from different plant species, residues G17 and N23, which are critical for REF1 interaction with its receptor [27–29], are highly conserved. We thus hypothesize the promoting effect of REF1 on transformation and regeneration efficiency in tomato can be used to improve *Agrobacterium*-mediated transformation and regeneration in citrus.

Four REF1 sequences were identified in *Citrus* species, differing at the 2^nd^, 4^th^, 6^th^, and 18^th^ amino acid positions. Specifically, *C. sinensis* is a hybrid between pomelo (*C. maxima*) and mandarin (*C. reticulata*) and contains two different REF1 peptides, differing by one amino acid. The first REF1 isoform RALRVIKRPPRPPGSSGGGGQIN was named as CsREF1–1, which was also found in *C. medica* and *C. limon.* The second isoform RALGVIKRPPRPPGSSGRGGQIN was named CsREF1–2, which was also detected in *C. maxima* and *C. aurantiifolia*. Both CsREF1–1 and CsREF1–2 were synthesized for testing their efficacy on *Agrobacterium*-mediated citrus transformation and regeneration. The tomato-derived SlREF1 peptide was included for comparison purposes.

### REF1 peptides enhance transformation efficiency and callus formation in *C. Sinensis*, but suppress shoot regeneration

**Figure 2:**
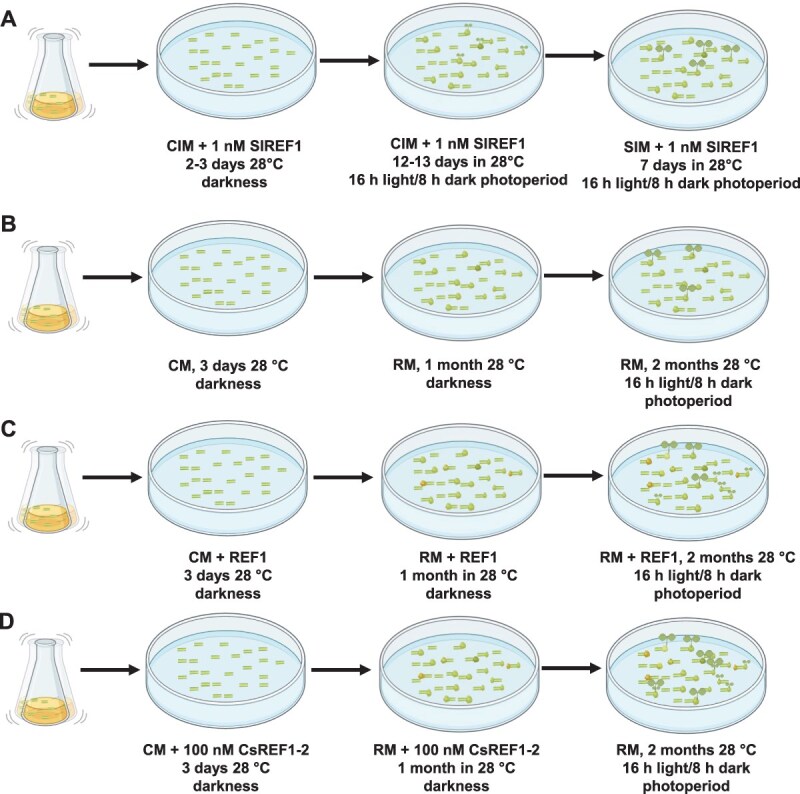
Schematic illustration of the citrus transformation and shoot regeneration with or without REF1 peptide in the medium. (A) Tomato transformation and shoot regeneration with REF1. For callus induction, 7-day-old tomato seedlings were cut with scissors and hypocotyl explants were incubated on CIM (MS medium supplemented with 3% sucrose, 0.8% Agar, 2 mg/L 2,4-D, 0.1 mg/L 6-BA) with 1 nM REF1 for 14 days. Afterwards, hypocotyl explants were transferred to SIM (MS medium supplemented with 3% sucrose, 0.8% Agar, 1 mg/L IAA, 1.75 mg/L ZT) with 1 nM REF1 for another 7 days for shoot regeneration. (B) Regular citrus transformation method without REF1. (C) Tested citrus transformation method with REF1. (D) Improved citrus transformation method with REF1 (100 nM) included in the RM medium in the first month but removed afterwards for shoot regeneration. CM: co-cultivation medium; RM: regeneration medium.

We next investigated the impact of REF1 on the transformation and shoot regeneration efficiency of **‘Hamlin’ sweet orange, a widely cultivated commercial variety**. For this experiment, we used *A. tumefaciens* strain **EHA105** carrying the **pCXH2-CmYLCV-Cas9-CsU6-tRNA** construct, with GFP signal selection maker and kanamycin screening marker, which is suitable for genome editing to transform the **‘Hamlin’** epicotyl explants [12]. The co-cultivation medium (CM) and regeneration medium (RM) were supplemented with **CsREF1–1, CsREF1–2, or SlREF1** peptides at final concentrations of **1 nM, 10 nM, or 100 nM,** with no REF1 supplemented as the control (Fig. 2). Following *Agrobacterium*-mediated transformation, the excised epicotyl explants were first placed on CM with or without REF1 peptides. **After three days, the explants were transferred to RM supplemented with the same peptide concentrations to promote shoot regeneration (****Fig.**  **2****).**

**Figure 3:**
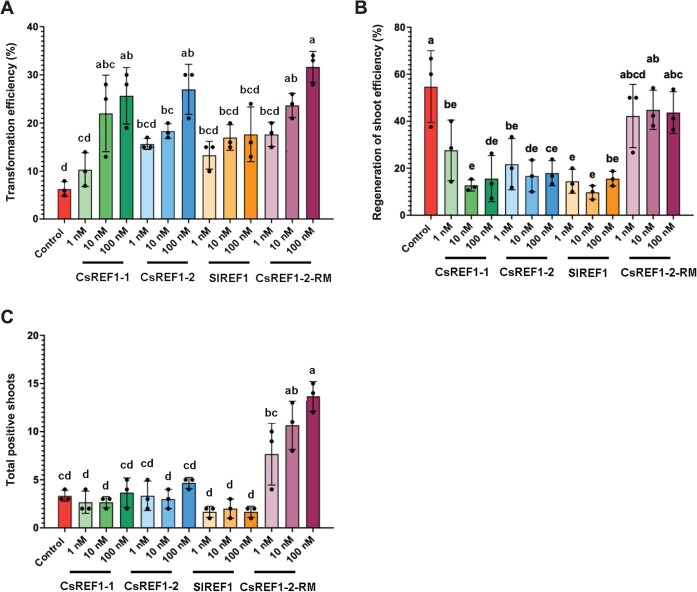
Effects of three different REF1 peptides on transformation, callus formation and shoot regeneration via *Agrobacterium*-mediated citrus epicotyl transformation. (A) Effects of REF1s (SlREF1, CsREF1–1, CsREF1–2) on citrus transformation efficiency, which was calculated 1 month after REF1 application. Transformation efficiency = No. of GFP positive callus/No. of transformed explants. (B) Effects of REF1s on citrus regeneration efficiency. The explants were kept on CM with or without REF1 for another 2 months for shoot regeneration. Efficiency of shoot regeneration = No. of GFP-positive shoots/No. of GFP positive callus. (C) CsREF1–2-RM treatment increases efficiency of total positive shoot formation. Efficiency of total positive shoot formation = No. of GFP-positive shoots/100 transformed epicotyls. The CsREF1–2-RM treatment consisted of CsREF1–2 in CM and RM only during the first month. The control consisted of CM and RM without REF1 supplementation (Fig. 2B). CsREF1–1, CsREF1–2, and SlREF1 treatments indicate continuous REF1 in CM and RM for 2 months (Fig. 2C). CsREF1–2-RM indicates CsREF1–2 was applied only during the first month in RM followed by one month on REF1-free RM (Fig. 2D). Data are presented as mean ± SD from three independent experiments (n = 100 per experiment). Different letters represent significant differences (P < 0.05, one-way ANOVA with Tukey’s HSD post-hoc test).

After one month of incubation in darkness, GFP positive calli were quantified to calculate the transformation efficacy which was calculated as: No of GFP-positive calli / number of transformed epicotyl explants. Compared to the negative control, CsREF1–1 at the final concentration of 10 nM and 100 nM, and CsREF1–2 at 100 nM significantly increased GFP positive callus formation and transformation efficiency compared with the control (Fig. 3A). However, SlREF1 at all three concentrations and CsREF1–1 (1 nM) and CsREF1–2 (1 nM and 10 nM) had no significant effect on transformation efficiency even though the treatments showed slight improvement but not statistically significant (P > 0.05). The most effective treatment was CsREF1–2 at 100 nM, which improved transformation efficiency by 4.3-fold, followed by CsREF1–1 at 100 nM, which increased 4.1-fold compared with the control (Fig. 3A).

**Figure 4:**
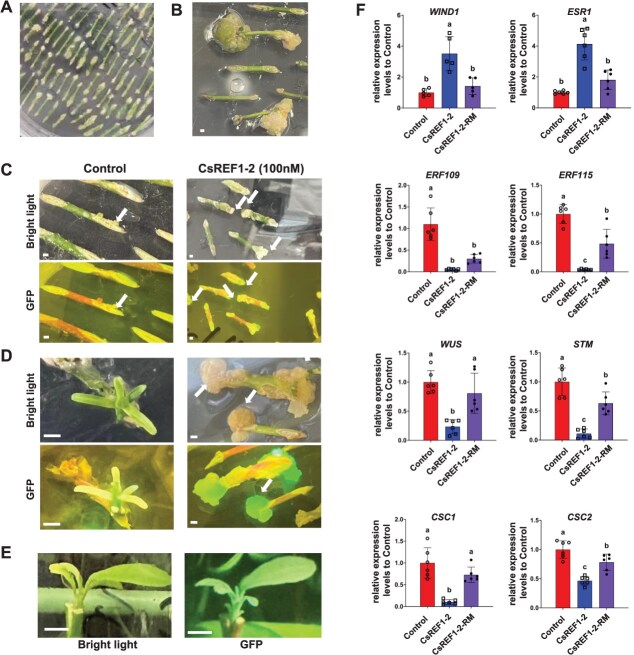
CsREF1–2 enhances citrus transformation efficiency by promotes callus proliferation during regeneration. (A) Callus regenerated with 100 nM CsREF1–2 treatment after one month. (B-C) Large clusters of callus formed with continuous 100 nM REF1–2 treatment after another 2 months on RM. (D) GFP-positive shoot regenerated with 3 months of 100 nM REF1–2-RM treatment. (E) Micrografting GFP-positive shoot onto Carrizo rootstock. Bar = 0.1 cm. (F) RT-qPCR analysis of expression of *WIND1*, *ESR1*, *ERF109*, *ERF115*, *WUS*, *STM*, *CSC1*, and *CSC2* in explant callus after 2 months of culture, with and with out CsREF1–2. *CsGAPDH* was used as an endogenous control for normalization. GAPDH: Glyceraldehyde-3-phosphate dehydrogenase. Data shown are mean ± SD, n = 6. Lowercase letters indicate significant differences among different lines (p < 0.05; Games-Howell post-hoc test). The experiment was repeated twice with similar results. The experiments were repeated at least two times with similar results. Bar = 0.1 cm.

Afterwards, GFP-positive calli were transferred to fresh RM containing CsREF1–1, CsREF1–2, or SlREF1 at the final concentration of 1, 10, and 100 nM and cultured under a 16 h light/8 h dark photoperiod at 28°C for 2 months (Fig. 2C). At the end of this period, the number of GFP-positive shoots regenerated from each treatment was quantified (Figs. 3B, 4 A-D). Efficiency of shoot regeneration was calculated as No. of GFP-positive shoots/No. of GFP positive callus. Intriguingly, we observed that REF1s had a negative impact on shoot regeneration in **‘Hamlin’** explants. The control without any REF1 supplement exhibited the highest shoot regeneration efficiency (54.72%) compared with the REF1 treatments (ranging from 11.8 to 27.6%) (Fig. 3B). The explants in RM with REF1 supplement mainly developed into large callus clusters rather than shoots **(****Fig.**  **4B****,**  **C****). Because of the opposite effect of REF1 on citrus transformation and regeneration, we further quantified the efficiency of** total positive shoot formation, which results from the combined effect of both factors. Efficiency of total positive shoot formation was calculated as No. of GFP-positive shoots/**100 transformed epicotyls. The efficiency of total positive shoot formation was 3.3% for the control, 1.3% for 1 nM SlREF1, 1.7% for 10 nM SlREF1, 2.3% for 100 nM SlREF1,** 2.7% for 1 nM CsREF1–1, 2.7% for 10 nM CsREF1–1, 3.7% for 100 nM CsREF1–1, 3.3% for 1 nM CsREF1–2, 3.0% for 10 nM CsREF1–2, and 4.7% for 100 nM CsREF1–2. Thus, although CsREF1–1 and CsREF1–2 markedly enhanced transformation efficiency, they had only a limited impact on the final recovery of GFP-positive shoots because both peptides negatively affected shoot regeneration. Notably, CsREF1–2 at 100 nM produced the most positive shoots, likely because its stronger enhancement of citrus transformation generated a larger pool of GFP-positive calli; despite reduced regeneration per callus, the greater number of transformed calli ultimately yielded more GFP-positive shoots.

To mitigate the negative effect of REF1 on shoot regeneration, we tested a new treatment in which citrus epicotyl explants were exposed to CsREF1–2 (final concentration at 1 nM, 10 nM, or 100 nM) in the CM during the first month and then transferred to RM without REF1 for regeneration (Fig. 2D). Compared to the 16.7–21.7% shoot regeneration efficacy for CsREF1–2 with the original procedure (2 months on RM with REF1), the efficiencies for the treatment without REF1at the later stage (one month on RM with REF1 and with REF1 removed afterwards) ranged from 42.2% to 44.9% (Figs. 2 and 3B). Moreover, treatment with CsREF1–2 at concentrations of 1, 10, and 100 nM without REF1 peptides at the later stage (Fig. 2D) resulted in improved efficiencies of total positive shoot formation of 7.7%, 10.7%, and 13.7%, respectively, significantly higher than both the no-treatment control and the corresponding treatments using the original procedure (Figs. 2C,D and 3C). Specifically, treatment with 100 nM CsREF1–2 under the optimized procedure generated 4.1-fold more total positive shoots than the non-treated control (Fig. 3C).

### CsREF1–2 peptide application differently affect expression of citrus genes involved in callus formation and shoot regeneration

To understand the different effects of different treatments of CsREF1 on shoot regeneration, we investigated expression of genes involved in callus formation and shoot regeneration with reverse transcription quantitative PCR (RT–qPCR). Specifically, we compared expression profiles of two-month-old calli in three culture conditions, control without REF1 peptide, continuous 100 nM CsREF1–2 (hereafter continuous REF1), and 100 nM CsREF1–2 applied only during the first month followed by one month on REF1-free medium (hereafter removal of REF1). The select genes include *WIND1*, *ENHANCER OF SHOOT REGENERATION1* (*ESR1*) [30], *ETHYLENE RESPONSE FACTOR 109* (*ERF109*) [31], *ETHYLENE RESPONSE FACTOR 115* (*ERF115*) [32], *WUSCHEL* (*WUS*) [33], *SHOOT MERISTEMLESS* (*STM*) [34], *CUP-SHAPED COTYLEDON 1* (*CSC1*), and *CUP-SHAPED COTYLEDON 2* (*CSC2*) [35]. Continuous CsREF1–2 exposure strongly induced *WIND1* and *ESR1* (Fig. 4F), consistent with REF1-promoted dedifferentiation and callus proliferation that contribute to increased transformation efficiency (Fig. 4C). In contrast, continuous REF1 treatment significantly reduced expression of *ERF115*, *WUS*, *STM*, *CSC1*, and *CSC2* compared with the non-treated control and removal of REF1 treatments (Fig. 4F). The removal of REF1 treatment also significantly down-regulated *ERF109*, *ERF115*, *STM*, *CSC2*, but not *WUS*, and *CSC1* compared with the non-treated control (Fig. 4F).

### Molecular analysis of transgenic plants

**Figure 5:**
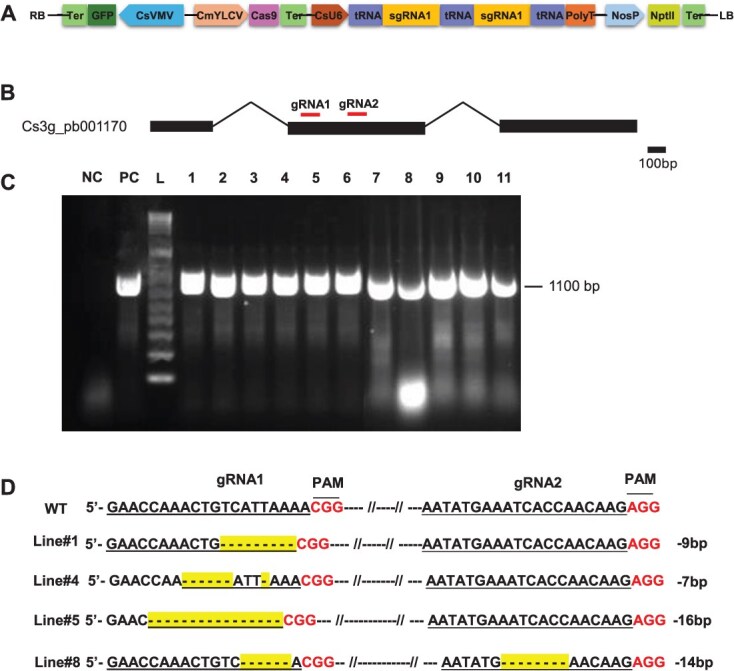
Validation of Cas9 activity in CsREF1–2 applied *Agrobacterium*-mediated citrus transformation. (A) Schematic diagram of the CRISPR/Cas construct used for genome editing. CsVMV, cassava vein mosaic virus promoter; CmYLCV, cestrum yellow leaf curling virus promoter; CsU6, citrus U6 promoter; Ter, terminator. For GFP, Nos terminator; for gRNA target, poly T terminator followed by HSP 18.2 terminator; for Cas9, HSP 18.2 terminator. RB, T-DNA right border; LB, T-DNA left border. (B) Gene structure of Cs3g_pb001170, two gRNAs are shown in red. (C) PCR amplification of Cas9 from the GFP lines. L: ladder; PC: pCXH2 plasmid as a positive control; 1–11: GFP positive lines. NC: Wild-type citrus DNA as a negative control. (D) The genotypes of the edited lines. Red letters indicate the PAM sequences; underlined letters indicate gRNA; yellow highlights indicate the mutated nucleotides.

To verify successful transformation and gene-editing events, transgenic plants were further characterized. Putative transgenic shoots were initially identified by the visible GFP fluorescence (Fig. 4C, D). Subsequently, genomic DNA was extracted from these GFP-positive transgenic plants to amplify the Cas9 coding sequence. The amplification band of 957 bp was detected in randomly selected GFP-positive shoots, confirming the integration of the Cas9 transgene into the plant genome (Fig. 5C). To further validate editing efficiency, the target genomic region amplified from 11 transgenic lines was analyzed by Sanger sequencing. Sequence analysis revealed four lines were edited, resulting in a 9 bp, 7 bp, 16 bp, and 14 bp deletions (Fig. 5D). The average editing efference was 36% similar as previously reported [12].

### Application of REF1 in transformation of *Carrizo citrange*, a citrus hybrid

To determine whether the effect of REF1 on citrus transformation and regeneration is conserved in other non-sweet orange species, we tested *Carrizo citrange*, a hybrid of Washington Navel orange (*C. sinensis*) and *Poncirus trifoliata*. For this test, explants were inoculated with *A. tumefaciens* carrying the Cas9 construct and transferred to CM supplemented with 100 nM CsREF1–2 peptide for one month and removed afterwards (Fig. 2D). The results showed that 100 nM CsREF1–2 increased callus formation by 2.8-fold and increased total positive shoots by 3.3-fold compared with the non-treated control (Supplementary Table S1), similar as sweet orange.

### Predication of the interaction of CsREF1–1, CsREF1–2, and SlREF1 with PORK1 using AlphaFold3 and PRODIGY

**Figure 6:**
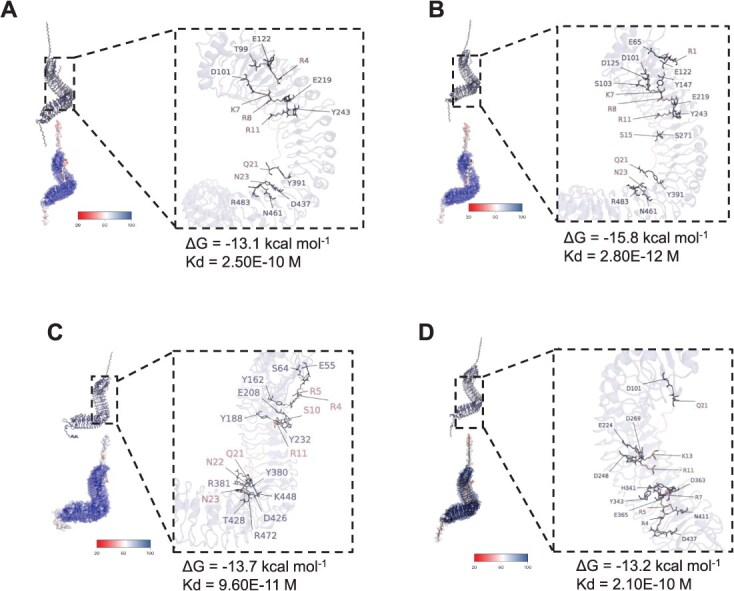
Predicted structures of PORK1 complexes with CsREF1–1, CsREF1–2, or SlREF1 by AlphaFold3. The AlphaFold3 model of the LRR extracellular domain of CsPORK1 with (A) CsREF1–1, and (B) CsREF1–2. Predicted binding sites were magnified and labelled. (C) The AlphaFold3 model of the LRR extracellular domain of SlPORK1 with SlREF1. (D) The AlphaFold3 model of the LRR extracellular domain of CsPORK1 with SlREF1. Gibbs free energy (ΔG) and corresponding dissociation constants (Kd) were calculated with the PRODIGY at 25°C. Color represents the predicted Local Distance Difference Test (pLDDT).

Because PORK1 is the receptor perceiving REF1 via physical interaction to induce callus formation [26], we hypothesize that the three REF1 CsREF1–1, CsREF1–2, and SlREF1 have different binding affinities with PORK1 since they perform differently in promoting citrus transformation. We next predicated their interactions with CsPORK1 using AlphaFold3. The CsREF1–1-CsPORK1, CsREF1–2–CsPORK1, and SlREF1–CsPORK1 complexes yielded ipTM values of 0.62, 0.54, and 0.48, respectively, whereas the SlREF1–SlPORK1 complex achieved the highest score of 0.85, consistent with the interactions detected using pulldown assay [26]. Because ipTM values below 0.5 often indicate unstable or incorrect binding modes [36], the SlREF1–CsPORK1 prediction may be less reliable and was removed from further analysis. Next, models with ipTM values greater than 0.5 were retained to predict binding affinity using PRODIGY [37]. The binding free energy (ΔG) values were − 15.8 kcal/mol for CsREF1–2-CsPORK1, −13.1 kcal/mol for CsREF1–1-CsPORK1, and − 13.7 kcal/mol for SlREF1-SlPORK1, indicating that CsREF1–2- CsPORK1 had the highest binding affinity (Fig. 6). Because lower ΔG reflects stronger interactions, these results suggest that CsREF1–2 forms the most stable complex with CsPORK1. This trend was further supported by the predicted dissociation constants (Kd), with lower Kd value indicating a stronger affinity. For CsPORK1–CsREF1–1, CsPORK1–CsREF1–2, and SlREF1-SlPORK1, Kd values were predicated to be 2.5 × 10^−10^ M, 2.8 × 10^−12^ M, and 9.6 × 10^−11^ M, respectively.

## Discussion

Citrus, one of the world’s most economically valuable fruit crops, is increasingly threatened by biotic and abiotic stresses, underscoring the urgency of genetic improvement via *Agrobacterium*-mediated transformation [3, 4]. However, this technology is severely constrained by the recalcitrance of woody perennials, rooted in multiple biological barriers [38, 39]. Unlike herbaceous model plants, citrus explants exhibit low cell division activity and limited totipotency, hindering the transition from differentiated cells to regenerable calli [2, 7, 40]. Their thick, lignified cell walls also act as physical barriers, impairing *Agrobacterium* attachment, colonization, and T-DNA transfer [41]. Strong genotype dependence further restricts applicability, as only a few citrus cultivars produce regenerable calli, while most commercial varieties remain highly resistant to in vitro transformation [40]. Traditional *Agrobacterium* transformation strategies have yielded limited, genotype-specific results, failing to provide a universal solution for all citrus species [16, 42, 43]. Against this backdrop, our study identified two citrus-derived REF1 peptides, uncovered their dual role in boosting transformation efficiency while inhibiting shoot regeneration, and developed a staged application procedure of CsREF1 peptides to promote *Agrobacterium*-mediated citrus transformation. Such a staged application of CsREF1–2 achieved a 4.1-fold increase in GFP-positive shoot efficiency in Hamlin sweet orange and a 3.3-fold increase in Carrizo citrange, with 36% CRISPR/Cas9 editing efficiency, offering a useful tool for citrus and other recalcitrant woody plants.

### Conservation and functional divergence of REF1 peptides across plant species

A striking feature of Pep/REF1 peptides across plant lineages is the conservation of key residues within the bioactive C-terminal domain, including the positions corresponding to G17 and the terminal N23 across multiple species. Classic structure activity studies of AtPep/REF1 and ZmPep/REF1 peptides family demonstrated that the G17 position is critical for elicitor activity and receptor engagement, and that removal of the terminal asparagine (N23) markedly reduces AtPep1 activity and its association with the AtPEPR1 receptor [27–29]. Consistent with their critical roles, comparative analyses demonstrate strict conservation of the glycine position across Pep homologs from diverse species, and alignments of the Arabidopsis Pep family reveal a highly conserved C-terminal motif SSG(x)₂G(x)₂N, supporting that the C-terminus contains the dominant determinants of Pep bioactivity [29, 44]. In our sequence analysis of Pep/REF1 homologs from multiple plant species spanning woody perennials and herbaceous annuals, we likewise observed conservation at G17 and N23, and within Rutaceae we identified a highly conserved C-terminal motif (RPPRxGSSGxGGxIN) similar to the Arabidopsis consensus (SSG(x)₂G(x)₂N), indicating that strong sequence conservation on the active C-terminal region is maintained in citrus relatives as well.

Functionally, these conserved sequence features align with a broadly conserved signaling pathway in which PEP-derived peptides act as local damage cues perceived by PEPR-family receptor kinases. In Arabidopsis, AtPep1 is recognized by the cell-surface receptor PEPR1, establishing the Pep–receptor paradigm for endogenous innate immune response and regeneration [45, 46]. Across diverse plant families, Pep peptides are widely distributed and act as conserved signals in damage-associated responses, supporting cross-lineage conservation of the Pep module even when individual Pep paralogs diversify [47]. A recent breakthrough study in tomato revealed REF1 acts as a local wound signal perceived by PORK1 to activate the wound-reprogramming regulator WIND1, thereby promoting wound-induced regeneration programs [26]. Downstream, WIND1 is well established as a central switch for wound-induced dedifferentiation and callus formation, and it can promote shoot regeneration by transcriptionally activating ESR1, providing a mechanistic bridge from wound perception to acquisition of regenerative competence [48–50].

Importantly, potential functional divergence is not limited to ligand recognition at the receptor level. It can also arise downstream of perception, where the same upstream signal is interpreted through different regeneration programs. In tomato, SlREF1 signaling activates SlWIND1 and supports both callus formation and shoot regeneration. In citrus, CsREF1 induced CsWIND1 upregulation is associated primarily with callus proliferation but does not robustly activate key shoot regeneration regulators (CSC1, CSC2, STM, and WUS), which limits conversion of callus to shoots. Together, these data suggest a model in which genus-specific differences in receptor compatibility and downstream regulatory wiring contribute to the distinct biological outcomes observed for REF1 peptides as exemplified by citrus and tomato. Gene family complexity likely reinforces this divergence. Both citrus and tomato encode multiple REF1/PEP1 which can generate distinct mature peptides that vary in sequence, processing efficiency, stability, and receptor preference [51]. Expansion and diversification of these paralogs provide additional opportunities for functionalization or neofunctionalization, further promoting lineage specific tuning of the REF1-PORK1 module and its developmental outputs [51, 52].

### Mechanistic basis of CsREF1’s dual function in citrus

Our data support a biphasic, stage-dependent model in which CsREF1 enhances early transformation output by accelerating wound-triggered cellular reprogramming and callus proliferation, but prolonged signaling antagonizes the transcriptional and hormonal state required for shoot organogenesis. In the first phase, CsREF1 application increases the pool of competent, actively dividing cells at the wound site, which is the window when *Agrobacterium* attachment, infection, and T-DNA integration are most productive. Consistent with this, CsREF1 treatments increased GFP-positive callus formation, with CsREF1–2 at 100 nM producing the strongest effect. This phenotype is consistent with the Pep/REF1–PEPR/PORK1–WIND1 framework described above, linking peptide perception to wound reprogramming and callus initiation. In citrus the downstream program diverges after callus induction. Although CsREF1 induced *CsWIND1* and *CsESR1*, continuous CsREF1 exposure suppressed shoot/meristem regulators such as *WUS* [33], *STM* [34], *CSC1*, and *CSC2* [35], providing a direct molecular explanation for the reduced shoot regeneration observed under prolonged peptide treatment. Removing the REF1 treatment recovers the imbalanced WIND1/ESR1 expression and eliminates the inhibition effect on expression of *WUS*, *STM*, *CSC1*, and *CSC2*, leading to shoot regeneration.

A plausible mechanistic interpretation is that persistent CsREF1 signaling maintains a wound-induced reprogramming state and biases the hormonal and transcriptional landscape toward wound and defense outputs that sustain proliferation and callus maintenance but are less permissive for shoot regeneration [53]. The WIND1 module is sufficient to promote dedifferentiation and callus initiation across species, yet efficient shoot regeneration typically requires additional permissive cues, most prominently cytokinin-promoting activity and activation of a shoot-meristem gene regulatory network [53–55]. Consistent with this general principle, ectopic AtWIND1 activity induces callus broadly in rapeseed, tobacco, and tomato, but robust shoot formation was only reported in tomato [56]. In tobacco, shoot formation can be improved by coupling wound-driven reprogramming with cytokinin biosynthesis, for example by co-expressing *ipt* (isopentenyltransferase) under control of the *ESR1* promoter with ectopic AtWIND1 activity, can induce cytokinin and facilitating organogenic progression [32]. Similarly, in chili peppers (*Capsicum annuum*), regeneration outcomes are strongly dependent on growth regulator context, and *C. annuum* REF1-associated callus induction and subsequent organogenesis occurs in conditions with optimized hormone combinations across stages, including callus induction, shoot formation or elongation, and rooting [57]. Collectively, these cross-species observations support the interpretation that REF1 or WIND1 signaling is relatively conserved for callus initiation, whereas progression from callus to shoots is constrained by species- and genotype-dependent bottlenecks in hormone responsiveness and shoot-meristem activation, limitations that are often more pronounced in woody species [58]. Therefore, improving our understanding of shoot meristem activation in woody species is essential for future advances in shoot regeneration, particularly to define how the timing and magnitude of WIND1 and ESR1 activation can be tuned to support organogenesis without locking tissues into a callus-maintenance state.

### Multifactorial causes for SlREF1 functional inactivation in citrus

SlREF1 did not significantly improve citrus transformation in our tested conditions. Several non-mutually exclusive explanations may account for SlREF1’s weak activity in citrus: limited peptide bioavailability in culture due to degradation, sequestration, or chemical inactivation; inefficient recognition by citrus PEPR/PORK1 or recognition by different PEPR, and associated co-receptors because of ligand–receptor divergence; and/or downstream signaling divergence that biases outputs toward defense or stress programs rather than WIND1/ESR1-driven reprogramming and regeneration [28, 59, 60]. In addition, SlREF1 may require a different dose, timing, or exposure window than tested here.

REF1 peptides and PEPR-like receptors are recognizable across plant lineages, sequence similarity alone does not guarantee functional equivalence [52]. This is because REF1 ligands and their cognate PORK1/PEPR receptors can undergo lineage-specific co-evolution, such that modest divergence in either the peptide or the receptor ectodomain can shift binding compatibility, signaling amplitude, and downstream transcriptional output [51, 52]. In line with this possibility, AlphaFold3 docking and PRODIGY estimates predicted a more reliable and relatively stable interaction between CsREF1 and CsPORK1, whereas the predicted SlREF1–CsPORK1 complex exhibited low interface confidence (ipTM = 0.48), consistent with unstable binding and altered recognition determinants in heterologous interactions [36, 61]. Together, these in silico analyses provide supporting, but not definitive, evidence for a perception-level barrier in cross-species ligand–receptor compatibility and offer a plausible mechanistic hypothesis for the weak activity of SlREF1 in citrus^2747^. More broadly, this is consistent with lineage-specific co-evolution of PEP/REF1 peptides and PEPR/PEPR-like receptors, which can produce interfamily incompatibility at the perception step [28, 47, 52, 62]. Such predictions need further experimental validations, e.g., yeast two-hybrid or Co-IP. Nevertheless, the predication provides a putative explanation for why heterologous REF1 peptides may show diminished performance in woody species.

### Implications, limitations and future perspectives

Beyond the mechanistic insight, CsREF1 application offers practical advantages relative to co-expression of regeneration-promoting genes, particularly in transformation-recalcitrant woody crops. Co-expression strategies often require adding additional expression cassettes, increasing plasmid size and T-DNA complexity, which can reduce transformation efficiency, especially when using large constructs such as standard Cas9 vectors [24, 63]. Moreover, regeneration regulators introduced by co-expression can confound functional analyses by altering developmental and stress-response pathways, complicating phenotype attribution to the target gene [64–66]. In contrast, exogenous peptide application can enhance transformation outputs without increasing vector size or permanently reprogramming gene expression, and it may be compatible with transgene-free editing workflows [67].

We also note workflow limitations that remain relevant for citrus deployment. The occurrence of escapes (non-transformed plants) likely reflects insufficient selection stringency, allowing non-transformed cells to survive and regenerate [68]. Kanamycin is widely used for citrus transformation [69,70],; here we used 100 mg/L kanamycin, comparable to or higher than some reports, yet escapes still occurred [65]. Increasing kanamycin further can damage explants and suppress regeneration [71], implying that more effective selectable markers or selection schemes may improve overall transformation stringency without exacerbating regeneration penalties [12, 72].

In summary, our study demonstrates citrus REF1 peptides as effective enhancers of early transformation output, while demonstrating that prolonged REF1 exposure reduces shoot regeneration. By integrating a staged REF1 application into the *Agrobacterium*-mediated transformation workflow for citrus epicotyls, we improved final GFP-positive shoot recovery, validated the approach across two genotypes, and enabled efficient CRISPR/Cas9 genome editing with 36% editing efficiency. These findings establish CsREF1–2 as a promising tool for citrus biotechnology and suggest that similar peptide-enabled strategies may be extendable to other woody, transformation-recalcitrant crops [73, 74].

## Materials and methods

### Seed germinated

*Carrizo citrange* seeds were obtained from Lyn Citrus Seeds (Arvin, CA, United States). Sweet orange (cv. Hamlin) seeds were collected from mature fruit grown at the Citrus Research and Education Center, University of Florida. Seed germination was conducted as described previously [70, 75]. Briefly, mature seeds were sterilized in a 30% (v/v) bleach solution for 10 minutes, followed by thorough rinsing. After five washes with sterile distilled water, seeds were transferred to 25 mm × 150 mm glass tubes containing 3 mL of MS medium (4.46 g/L Murashige & Skoog Basal Medium with vitamins (PhytoTech labs M519) with 3% sucrose, 0.3%Gelzan™, pH 5.8) for 1 month under dark and 1–2 month with 16 h light/8 h dark photoperiod at 28°C.

### Citrus regeneration and transformation assays

To evaluate the transformation and regeneration efficiency of REF1, we employed the pCXH2-CmYLCV-Cas9-CsU6-tRNA construct [12]. In brief, for the targeted gene two specific gRNAs were designed using CRISPR-P (http://crispr.hzau.edu.cn/cgi-bin/CRISPR2/CRISPR#) and assembled into vectors by Golden Gate cloning. The gRNA cassette was excised from pXH1-CmYLCV-targetgrna using FspI and XbaI and inserted into the ZraI/XbaI site of the binary vector pCXH2 to generate the final constructs pCXH2-CmYLCV-targetgrna (primers in Supplementary Table S2). The final construct was introduced into *A. tumefaciens* strain EHA105. This binary vector contained a **GFP reporter gene** for selection of positive transformation and regeneration events.

Hamlin sweet orange epicotyls were used for *Agrobacterium*-mediated transformation as described previously with modifications [70, 75]. Briefly *Agrobacterium*-transformed epicotyls were placed on the co-cultivation medium (CM, MS medium supplemented with 2 mg/L 6-benzylaminopurine, 0.5 mg/l indole-3-acetic acid, 1 mg/l 2,4-dichlorophenoxyacetic acid, 100 uM/L Acetosyringone) containing three REF1 peptide of (SlREF1, CsREF1–1, and CsREF1–2) at four different concentrations (0 nM, 1 nM, 10 nM, and 100 nM) for 3 days in the dark. Subsequently, the explants were transferred to the regeneration medium (RM, MS medium supplemented with 2 mg/L 6-benzylaminopurine, 0.5 mg/l indole-3-acetic acid, pH 5.8, 100 mg/L kanamycin, 350 mg/L timentin) containing the same concentrations and REF1s. The explants were incubated in the dark for one month, followed by two months under a 16 h light/8 h dark cycle at 28°C for shoot regeneration.

The regenerated shoots were subsequently micro-grafted onto *Carrizo citrange* rootstock seedlings. The *Carrizo citrange* rootstock seedlings were geminated from seeds and were approximately 3 weeks old after germination. Transformation efficiency was calculated as No. of GFP-positive callus/No. of inoculated explants. Regeneration of shoot efficiency = No. of PCR-positive plants/No. of GFP positive calli. The experiments were conducted three times, each experiment consisting of 100 explants per treatment.

To further evaluate REF1 treatment, infected Hamlin epicotyls were placed on CM with 100 nM CsREF1–2 peptide for three days in the dark. Explants were then transferred to RM containing 100 nM CsREF1–2 peptide for one month in the dark, followed by transfer to RM without REF1 for two additional months under a 16 h light/8 h dark photoperiod at 28°C, with monthly subculture onto fresh RM. The experiments were conducted three times, each time consisting of 100 explants per treatment.

For *Carrizo citrange* transformation, infected epicotyls were treated using the same protocol (100 nM CsREF1–2, three days in CM, one month in RM with REF1, then transfer to RM without REF1).

### Transformation efficiency, shoot regeneration efficiency and Total positive shoots statistics analysis

Calculated based on 100 explants per experiment. After one month of regeneration medium (RM) culture, the number of GFP-positive calli was counted. Transformation efficacy was calculated as: No of GFP-positive calli / number of transformed epicotyl explants. After 3 month of culturing, positive transgenic shoots were first identified by GFP fluorescence, then excised for genomic DNA extraction using the CTAB method [63], followed by PCR amplification of a GFP fragment to confirm transformation. Efficiency of shoot regeneration was calculated as No. of GFP-positive shoots/No. of GFP positive callus. Efficiency of total positive shoot formation was calculated as No. of GFP-positive shoots/100 transformed epicotyls.

### Genotyping of citrus transformants

Positive transformants were initially identified by GFP fluorescence using a GFP filter and by selection on kanamycin-containing medium. Citrus genomic DNA was extracted using the cetyltrimethylammonium bromide (CTAB) method [76]. Positive transformants were further confirmed by Cas9-specific PCR amplification (Supplementary Table S2). Editing efficiency was assessed by amplifying the target region using Q5 High-Fidelity DNA polymerase (New England Biolabs, Ipswich, MA, USA), followed by cloning of PCR products and Sanger sequencing.

### Gene expression assays using reverse transcription quantitative PCR (RT-qPCR)

Total RNA was extracted using the RNeasy Plant Mini Kit (Cat. #74904, Qiagen), according to manufacturer’s instructions. cDNA was synthesized with Quantitect Reverse Transcription Kit (Cat. #205311, Qiagen) according to manufacturer’s instructions and diluted 10 times for RT-qPCR. Reactions were carried out by adding 1 μL of cDNA, 1 μL of each specific primer, 7 μL water and 10 μL Fast SYBR Green Master Mix (Cat. #4385617, Thermo-Fisher Scientific, Waltham, MA, USA) performed with QuantiStudio3 (Thermo-Fisher) using the standard fast protocol of 95°C for 20 s followed by 40 cycles of 95°C for 1 s and 60°C for 20 s. Denaturation protocol consisted of 95°C for 1 s, 60°C for 20 s and a final dissociation step of 95°C. Relative gene expression was calculated using the 2^-∆∆CT^ method [77]. *Glyceraldehyde-3-phosphate dehydrogenase (GAPDH)* gene was used as an endogenous control.

### Structural modelling and visualization

For the protein complexes, the LRR extracellular domains of CsPORK1 with CsREF1–1, CsREF1–2, and SlREF1 peptides, as well as SlPORK1 with the SlREF1 peptide, were predicted using AlphaFold3. Models with ipTM values greater than 0.5 were retained for further analysis.

To further examine the interaction between CsPORK1 and the SlREF1 peptide, individual structures were first predicted using AlphaFold3, and models with ipTM values greater than 0.7 were selected. Hydrogen bonds were defined as donor–acceptor pairs within 3.6 Å, while salt bridges were defined as interactions between oppositely charged groups within 5 Å. All protein structures were visualized and analyzed using PyMOL (v.2.5.2). Prediction of binding affinity in biological complexes analyzed using PRODIGY [37].

## Supplementary Material

Web_Material_uhag134

## Data Availability

The data supporting the results reported in the article can be found in this article and the supplementary data.

## References

[1] Talon M, Gmitter FG. Citrus genomics. Int J Plant Genomics. 2008;2008:1–17 10.1155/2008/528361 PMC2396216 18509486

[2] Liu S, Xu Y, Yang K. et al. Origin and de novo domestication of sweet orange. Nat Genet. 2025;57:754–62 40045092 10.1038/s41588-025-02122-4 PMC11906365

[3] Keneni G, Bekele E, Imtiaz M. et al. Genetic vulnerability of modern crop cultivars: causes, mechanism and remedies. Int J Plant Res. 2012;2:69–79

[4] Acevedo M, Pixley K, Zinyengere N. et al. A scoping review of adoption of climate-resilient crops by small-scale producers in low- and middle-income countries. Nat Plants. 2020;6:10 10.1038/s41477-020-00783-z PMC7553851 33051616

[5] Timmer LW, Garnsey SM, Graham JH. Compendium of Citrus Diseases, Second Edition | Diseases and Pests Compendium Series. The American Phytopathological Society; 2000:

[6] Rafie-Rad Z, Moradkhani M, Golchin A. et al. Abiotic Stresses Management in Citrus. In: Advances in Citrus Production and Research. IntechOpen, 2022,

[7] Wang X, Xu Y, Zhang S. et al. Genomic analyses of primitive, wild and cultivated citrus provide insights into asexual reproduction. Nat Genet. 2017;49:5 10.1038/ng.3839 28394353

[8] Koltunow AM, Hidaka T, Robinson SP. Polyembryony in citrus (accumulation of seed storage proteins in seeds and in embryos cultured in vitro). Plant Physiol. 1996;110:599–609 8742336 10.1104/pp.110.2.599 PMC157756

[9] Mieko O, Shimada T. Citrus breeding, genetics and genomics in Japan. Breed Sci. 2016;66:3–17 27069387 10.1270/jsbbs.66.3 PMC4780800

[10] Ravelonandro M, Briard P, Scorza R. et al. Robust response to plum pox virus infection via plant biotechnology. Genes. 2021;12:10.3390/genes12060816 PMC8227089 34071769

[11] Dutt M, El-Mohtar CA, Wang N. Biotechnological Approaches for the Resistance to Citrus Diseases. In: The Citrus Genome. Springer International Publishing: Cham, 2020,

[12] Huang X, Wang Y, Wang N. Frontiers | highly efficient generation of canker-resistant sweet Orange enabled by an improved CRISPR/Cas9 system. Front Plant Sci. 2022;12:10.3389/fpls.2021.769907 PMC8787272 35087548

[13] Wheatley MS, Yang Y. Versatile applications of the CRISPR/Cas toolkit in plant pathology and disease management. Phytopathology. 2021;111:1080–90 33356427 10.1094/PHYTO-08-20-0322-IA

[14] Cervera M, JoséJuárez AN. et al. Genetic transformation and regeneration of mature tissues of woody fruit plants bypassing the juvenile stage. Transgenic Res. 1998;7:1

[15] Fagoaga C, López C, Moreno P. et al. Viral-like symptoms induced by the ectopic expression of the p23 gene of citrus tristeza virus are citrus specific and do not correlate with the pathogenicity of the virus. Strain. 2007;18:435–45 10.1094/MPMI-18-0435 15915642

[16] Conti G, Xoconostle-Cázares B, Marcelino-Pérez G. et al. Citrus genetic transformation: an overview of the current strategies and insights on the new emerging technologies. Front Plant Sci. 2021;12:10.3389/fpls.2021.768197 PMC8670418 34917104

[17] Miyata LY, Harakava R, Stipp LCL. et al. GUS expression in sweet oranges (Citrus sinensis L. Osbeck) driven by three different phloem-specific promoters. Plant Cell Rep. 2012;31:2005–13 22801867 10.1007/s00299-012-1312-2

[18] Reyes CA, de Francesco A, Peña EJ. et al. Resistance to citrus psorosis virus in transgenic sweet orange plants is triggered by coat protein–RNA silencing. J Biotechnol. 2011;151:151–8 21084056 10.1016/j.jbiotec.2010.11.007

[19] Jia H, Wang N. Targeted genome editing of sweet Orange using Cas9/sgRNA. PLoS One. 2014;9:10.1371/journal.pone.0093806 PMC3977896 24710347

[20] Jia H, Orbovic V, Jones JB. et al. Modification of the PthA4 effector binding elements in type I CsLOB1 promoter using Cas9/sgRNAto produce transgenic Duncan grapefruit alleviating XccΔpthA4:dCsLOB1.3 infection. Plant Biotechnol J. 2016;14:1291–301 27071672 10.1111/pbi.12495 PMC11389130

[21] Jia H, Zhang Y, Orbović V. et al. Genome editing of the disease susceptibility gene CsLOB1 in citrus confers resistance to citrus canker. Plant Biotechnol J. 2017;15:817–23 27936512 10.1111/pbi.12677 PMC5466436

[22] Zhang F, LeBlanc C, Irish VF. et al. Rapid and efficient CRISPR/Cas9 gene editing in citrus using the YAO promoter. Plant Cell Rep. 2017;36:1883–7 28864834 10.1007/s00299-017-2202-4

[23] Jia H, Wang N. Generation of homozygous canker-resistant citrus in the T0 generation using CRISPR-SpCas9p. Plant Biotechnol J. 2020;18:1990–2 32167662 10.1111/pbi.13375 PMC7540605

[24] Xi J, Patel M, Dong S. et al. Acetosyringone treatment duration affects large T-DNA molecule transfer to rice callus. BMC Biotechnol. 2018;18:48 30092808 10.1186/s12896-018-0459-5 PMC6085696

[25] Wu H, Awan FS, Vilarinho A. et al. Transgene integration complexity and expression stability following biolistic or agrobacterium-mediated transformation of sugarcane. In Vitro Cell Dev Biol - Plant. 2015;51:603–11

[26] Yang W, Zhai H, Wu F. et al. Peptide REF1 is a local wound signal promoting plant regeneration. Cell. 2024;187:3024–3038.e14 38781969 10.1016/j.cell.2024.04.040

[27] Poretsky E, Dressano K, Weckwerth P. et al. Differential activities of maize plant elicitor peptides as mediators of immune signaling and herbivore resistance. Plant J. 2020;104:1582–602 33058410 10.1111/tpj.15022

[28] Tang J, Han Z, Sun Y. et al. Structural basis for recognition of an endogenous peptide by the plant receptor kinase PEPR1. Cell Res. 2014;25:110–20 25475059 10.1038/cr.2014.161 PMC4650589

[29] Pearce G, Yamaguchi Y, Munske G. et al. Structure–activity studies of AtPep1, a plant peptide signal involved in the innate immune response. Peptides. 2008;29:2083–9 18824048 10.1016/j.peptides.2008.08.019

[30] Lee K, Yoon H, Park OS. et al. ENHANCER OF SHOOT REGENERATION1 promotes de novo root organogenesis after wounding in Arabidopsis leaf explants. Plant Cell. 2024;36:2359–74 38445764 10.1093/plcell/koae074 PMC11132873

[31] Cai XT, Xu P, Zhao PX. et al. Arabidopsis ERF109 mediates cross-talk between jasmonic acid and auxin biosynthesis during lateral root formation. Nat Commun. 2014;5:5833 25524530 10.1038/ncomms6833

[32] Heyman J, Cools T, Canher B. et al. The heterodimeric transcription factor complex ERF115-PAT1 grants regeneration competence - PubMed. Nat Plants. 2016;2:16165 27797356 10.1038/nplants.2016.165

[33] Ikeda M, Mitsuda N, Ohme-Takagi M. Arabidopsis WUSCHEL is a bifunctional transcription factor that acts as a repressor in stem cell regulation and as an activator in floral patterning. Plant Cell. 2009;21:3493–505 19897670 10.1105/tpc.109.069997 PMC2798335

[34] Scofield S, Dewitte W, Nieuwland J. et al. The Arabidopsis homeobox gene SHOOT MERISTEMLESS has cellular and meristem-organisational roles with differential requirements for cytokinin and CYCD3 activity. Plant J. 2013;75:53–66 23573875 10.1111/tpj.12198

[35] Takada S, Hibara K, Ishida T. et al. The CUP-SHAPED COTYLEDON1 gene of Arabidopsis regulates shoot apical meristem formation. Development. 2001;128:1127–35 11245578 10.1242/dev.128.7.1127

[36] Abramson J, Adler J, Dunger J. et al. Accurate structure prediction of biomolecular interactions with AlphaFold 3. Nature. 2024;630:8016 10.1038/s41586-024-07487-w PMC11168924 38718835

[37] Honorato RV, Koukos PI, Jiménez-García B. et al. Structural biology in the clouds: the WeNMR-EOSC ecosystem. Front Mol Biosci. 2021;8:10.3389/fmolb.2021.729513 PMC8356364 34395534

[38] Dudareva N, Negre F, Nagegowda DA. et al. Plant volatiles: recent advances and future perspectives. Crit Rev Plant Sci. 2006;25:417–40

[39] Wang Y, Wang JH, Guo PR. et al. Developmental regulatory genes in recalcitrant forest trees: advances in somatic regeneration and genetic transformation. Front Plant Sci. 2025;16:10.3389/fpls.2025.1689705 PMC12661428 41323333

[40] Poles L, Licciardello C, Distefano G. et al. Recent advances of In vitro culture for the application of new breeding techniques in citrus. Plants. 2020;9:10.3390/plants9080938 PMC7465985 32722179

[41] Matthysse AG . Attachment of agrobacterium to plant surfaces. Front Plant Sci. 2014;5:10.3389/fpls.2014.00252 PMC4046570 24926300

[42] Donmez D, Simsek O, Izgu T. et al. Genetic transformation in citrus. Sci World J. 2013;2013:10.1155/2013/491207 PMC3745968 23983635

[43] Moniruzzaman M, Zhong Y, Huang Z. et al. Citrus cell suspension culture establishment, maintenance, efficient transformation and regeneration to complete transgenic plant. Plants. 2021;10:10.3390/plants10040664 PMC8066040 33808465

[44] Cui J, Sa E, Wei J. et al. The truncated peptide AtPEP1(9–23) has the same function as AtPEP1(1–23) in inhibiting primary root growth and triggering of ROS burst. Antioxidants. 2024;13:10.3390/antiox13050549 PMC11117541 38790654

[45] Pearce G, Yamaguchi Y, Munske G. et al. Structure-activity studies of AtPep1, a plant peptide signal involved in the innate immune response. Peptides. 2008;29:2083–9 18824048 10.1016/j.peptides.2008.08.019

[46] Yamaguchi Y, Pearce G, Ryan CA. The cell surface leucine-rich repeat receptor for AtPep1, an endogenous peptide elicitor in Arabidopsis, is functional in transgenic tobacco cells. Proc Natl Acad Sci USA. 2006;103:10104–9 16785433 10.1073/pnas.0603729103 PMC1502513

[47] Huffaker A, Pearce G, Veyrat N. et al. Plant elicitor peptides are conserved signals regulating direct and indirect antiherbivore defense. Proc Natl Acad Sci. 2013;110:5707–12 23509266 10.1073/pnas.1214668110 PMC3619339

[48] Iwase A, Ohme-Takagi M, Sugimoto K. WIND1: a key molecular switch for plant cell dedifferentiation. Plant Signal Behav. 2011;6:1943–5 22112447 10.4161/psb.6.12.18266 PMC3337183

[49] Iwase A, Harashima H, Ikeuchi M. et al. WIND1 promotes shoot regeneration through transcriptional activation of ENHANCER OF SHOOT REGENERATION1 in Arabidopsis. Plant Cell. 2017;29:54–69 28011694 10.1105/tpc.16.00623 PMC5304349

[50] Iwase A, Mitsuda N, Koyama T. et al. The AP2/ERF transcription factor WIND1 controls cell dedifferentiation in Arabidopsis. Curr Biol. 2011;21:508–14 21396822 10.1016/j.cub.2011.02.020

[51] Yamaguchi Y, Huffaker A, Bryan AC. et al. PEPR2 is a second receptor for the Pep1 and Pep2 peptides and contributes to defense responses in Arabidopsis. Plant Cell. 2010;22:508–22 20179141 10.1105/tpc.109.068874 PMC2845411

[52] Lori M, van Verk MC, Hander T. et al. Evolutionary divergence of the plant elicitor peptides (peps) and their receptors: interfamily incompatibility of perception but compatibility of downstream signalling. J Exp Bot. 2015;66:5315–25 26002971 10.1093/jxb/erv236 PMC4526913

[53] Iwase A, Harashima H, Ikeuchi M. et al. WIND1 promotes shoot regeneration through transcriptional activation of ENHANCER OF SHOOT REGENERATION1 in Arabidopsis. Plant Cell. 2017;29:54–69 28011694 10.1105/tpc.16.00623 PMC5304349

[54] Raspor M, Motyka V, Kaleri AR. et al. Integrating the roles for Cytokinin and auxin in De novo shoot organogenesis: from hormone uptake to signaling outputs. Int J Mol Sci. 2021;22:10.3390/ijms22168554 PMC8395325 34445260

[55] Ikeuchi M, Iwase A, Rymen B. et al. Wounding triggers callus formation via dynamic hormonal and transcriptional changes. Plant Physiol. 2017;175:1158–74 28904073 10.1104/pp.17.01035 PMC5664475

[56] Iwase A, Mitsuda N, Ikeuchi M. et al. Arabidopsis WIND1 induces callus formation in rapeseed, tomato, and tobacco. Plant Signal Behav. 2013;8:e27432 24389814 10.4161/psb.27432 PMC4091253

[57] Naeem B, Shams S, Ma L. et al. An optimized regeneration protocol for chili peppers (*Capsicum annuum* L.) through genotypespecific explant and growth regulator combinations. Seed Biol. 2025;4:0

[58] Iwase A, Kondo Y, Laohavisit A. et al. WIND transcription factors orchestrate wound-induced callus formation, vascular reconnection and defense response in Arabidopsis. New Phytol. 2021;232:734–52 34375004 10.1111/nph.17594 PMC9291923

[59] Royek S, Bayer M, Pfannstiel J. et al. Processing of a plant peptide hormone precursor facilitated by posttranslational tyrosine sulfation. Proc Natl Acad Sci. 2022;119:e2201195119 35412898 10.1073/pnas.2201195119 PMC9169856

[60] Corpo DD, Coculo D, Greco M. et al. Pull the fuzes: processing protein precursors to generate apoplastic danger signals for triggering plant immunity. Plant Commun. 2024;5:100931 38689495 10.1016/j.xplc.2024.100931 PMC11371470

[61] Xue LC, Rodrigues JP, Kastritis PL. et al. PRODIGY: a web server for predicting the binding affinity of protein–protein complexes. Bioinformatics. 2016;32:3676–8 27503228 10.1093/bioinformatics/btw514

[62] Ruiz C, Nadal A, Montesinos E. et al. Novel Rosaceae plant elicitor peptides as sustainable tools to control Xanthomonas arboricola pv. Pruni in Prunus spp. Mol Plant Pathol. 2018;19:418–31 28056495 10.1111/mpp.12534 PMC6638028

[63] Xing H-L, Dong L, Wang ZP. et al. A CRISPR/Cas9 toolkit for multiplex genome editing in plants. BMC Plant Biol. 2014;14:327 25432517 10.1186/s12870-014-0327-y PMC4262988

[64] Boutilier K, Offringa R, Sharma VK. et al. Ectopic expression of BABY BOOM triggers a conversion from vegetative to embryonic growth. Plant Cell. 2002;14:1737–49 12172019 10.1105/tpc.001941 PMC151462

[65] Hoerster G, Wang N, Ryan L. et al. Use of non-integrating Zm-Wus2 vectors to enhance maize transformation. In Vitro Cell Dev Biol - Plant. 2020;56:265–79

[66] Yu J, Deng S, Huang H. et al. Exploring the potential applications of the noninvasive reporter gene RUBY in plant genetic transformation. Forests. 2023;14:

[67] Su H, Wang Y, Xu J. et al. Generation of the transgene-free canker-resistant Citrus sinensis using Cas12a/crRNA ribonucleoprotein in the T0 generation. Nat Commun. 2023;14:3957 37402755 10.1038/s41467-023-39714-9 PMC10319737

[68] Ghorbel R, Juárez J, Navarro L. et al. Green fluorescent protein as a screenable marker to increase the efficiency of generating transgenic woody fruit plants. Theor Appl Genet. 1999;99:350–8

[69] Bekalu ZE, Panting M, Holme IB. et al. Opportunities and challenges of In vitro tissue culture systems in the era of crop genome editing. Int J Mol Sci. 2023;24:10.3390/ijms241511920 PMC10419073 37569295

[70] Huang X, Wang Y, Xu J. et al. Development of multiplex genome editing toolkits for citrus with high efficacy in biallelic and homozygous mutations. Plant Mol Biol. 2020;104:297–307 32748081 10.1007/s11103-020-01043-6

[71] Ali S, Mannan A, Oirdi ME. et al. Agrobacterium-mediated transformation of rough lemon (Citrus jambhiri lush) with yeast HAL2 gene. BMC Res Notes. 2012;5:10.1186/1756-0500-5-285 PMC3507645 22691292

[72] Huang X, Jia H, Xu J. et al. Transgene-free genome editing of vegetatively propagated and perennial plant species in the T0 generation via a co-editing strategy. Nat Plants. 2023;9:1591–7 37723203 10.1038/s41477-023-01520-y

[73] Li T, Jiang Z, Zhang L. et al. Apple (Malus domestica) MdERF2 negatively affects ethylene biosynthesis during fruit ripening by suppressing MdACS1 transcription. Plant J. 2016;88:735–48 27476697 10.1111/tpj.13289

[74] Penna S, Jain SM, Penna S. et al. Fruit crop improvement with genome editing, *In vitro and transgenic approaches*. Horticulturae. 2023;9:

[75] Jia H, Zou X, Orbovic V. et al. Genome editing in citrus tree with CRISPR/Cas9. Methods Mol Biol. 2019; 10.1007/978-1-4939-8991-1_17 30610640

[76] Pandey SS, Wang N. Targeted early detection of citrus Huanglongbing causal agent ' Candidatus Liberibacter asiaticus' before symptom expression - PubMed. Phytopathology. 2019;109:952–9 30667340 10.1094/PHYTO-11-18-0432-R

[77] Livak KJ, Schmittgen TD. Analysis of relative gene expression data using real-time quantitative PCR and the 2(-Delta Delta C(T)) method - PubMed. Methods. 2001;25:402–8 11846609 10.1006/meth.2001.1262

