# Supplementary material for: REF1 peptides significantly enhance transformation efficiency but inhibit shoot regeneration in citrus agrobacterium-mediated transformation
Source: Hortic Res. 2026 Apr 7;13(8):uhag134. doi: 10.1093/hr/uhag134 (PMC13438602; doi:10.1093/hr/uhag134)
Supplement: Supplementary file 1 [file web_material_uhag134.zip › supplementary.pdf]

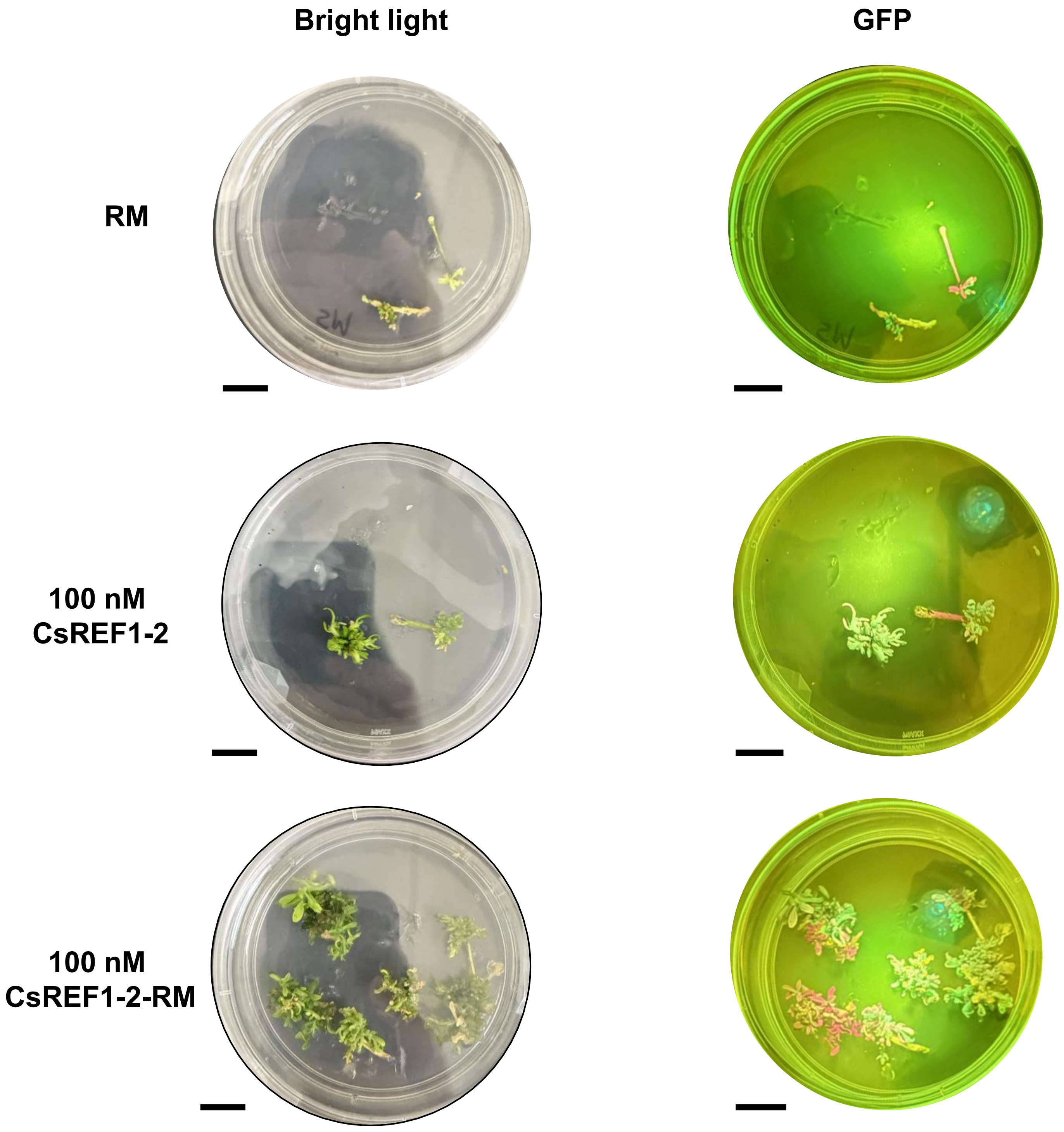

Supplementary Figure 1. Effects of different REF1 variants on transformation and callus formation via *Agrobacterium*-mediated citrus epicotyl transformation. Pictures shown are three months after *Agrobacterium*-mediated transformation. Typical GFP-positive shoot from the RM without REF1, 100 nM CsREF1-2 following the protocol shown in Fig. 2C, and 100 nM CsREF1-2-RM following the protocol shown in Fig. 2D. Bar=1cm
